# Attenuation of TNF-α-Induced Inflammatory Injury in Endothelial Cells by Ginsenoside Rb1 via Inhibiting NF-κB, JNK and p38 Signaling Pathways

**DOI:** 10.3389/fphar.2017.00464

**Published:** 2017-08-03

**Authors:** Ping Zhou, Shan Lu, Yun Luo, Shan Wang, Ke Yang, Yadong Zhai, Guibo Sun, Xiaobo Sun

**Affiliations:** ^1^Institute of Medicinal Plant Development, Peking Union Medical College and Chinese Academy of Medical Sciences Beijing, China; ^2^Beijing Key Laboratory of Innovative Drug Discovery of Traditional Chinese Medicine (Natural Medicine) and Translational Medicine Beijing, China; ^3^Key Laboratory of Bioactive Substances and Resource Utilization of Chinese Herbal Medicine, Ministry of Education Beijing, China; ^4^Key Laboratory of Efficacy Evaluation of Chinese Medicine against Glycolipid Metabolism Disorder Disease, State Administration of Traditional Chinese Medicine Beijing, China

**Keywords:** atherosclerosis, tumor necrosis factor-α, inflammation, ginsenoside Rb1, apoptosis

## Abstract

It is currently believed that inflammation plays a central role in the pathophysiology of atherosclerosis. Oxidative stress and redox-sensitive transcription factors are implicated in the process. Ginsenoside Rb1, a major active ingredient in processed Radix notoginseng, has attracted widespread attention because of its potential to improve cardiovascular function. However, the effects of ginsenoside Rb1 on tumor necrosis factor-α (TNF-α)-induced vascular endothelial cell injury and the underlying molecular mechanisms have never been studied. This study showed that TNF-α-induced oxidative stress, inflammation and apoptosis in human umbilical vein endothelial cells (HUVECs) could be attenuated by ginsenoside Rb1 pretreatment. Using JC-1, Annexin V/PI and TUNEL staining, and a caspase-3 activity assay, we found that Rb1 provided significant protection against TNF-α-induced cell death. Furthermore, Rb1 pretreatment could inhibit TNF-α-induced ROS and MDA production; increase the activities of SOD, CAT, and GSH-Px; and decrease the levels of IL-1β, IL-6, VCAM-1, ICAM-1, VEGF, MMP-2 and MMP-9. Importantly, the cytoprotective effects of Rb1 were correlated with NF-κB signaling pathway inhibition. Additionally, we found that Rb1 may suppress the NF-κB pathway through p-38 and JNK pathway activation, findings supported by the results of our experiments involving anisomycin (AM), a JNK and p38 activator. In conclusion, this study showed that ginsenoside Rb1 protects HUVECs from TNF-α-induced oxidative stress and inflammation by inhibiting JNK and p38. This inhibition suppressed NF-κB signaling and down-regulated the expression of inflammatory factors and apoptosis-related proteins.

## Introduction

Atherosclerosis (AS)-related cardiovascular disease is a major cause of death and disability worldwide (LW, Amp, and Wilkins, 2017). Accumulating evidence indicates that inflammation-induced endothelial dysfunction and apoptosis are pivotal triggers of atherosclerotic vascular disease (Friedman et al., 2005; Madhur et al., 2011). Tumor necrosis factor-α (TNF-α), a pivotal cytokine in the inflammatory cascade, has been reported to trigger interactions between invading monocytes and vascular endothelial cells, which subsequently induce endothelial apoptosis in the circulation (Jia et al., 2015). Consistent with these findings, the results of human studies suggest that TNF-α induces significant extracellular matrix deposition in the arterial wall, as well as several subsequent intracellular signaling events that ultimately increase the expression of the chemokines IL-6 and MCP-1 and the adhesion molecules VCAM-1, ICAM-1, and E-selectin (Yang et al., 2005; Chen P.-J. et al., 2016). A large body of evidence suggests that the TNF-α signaling cascade may lead to oxidative stress by escalating ROS production (Kim et al., 2010), which in turn mediates transcription factor activity. Activation of NF-κB is essential for the production of circulating and/or local vascular TNF-α, as well as adhesion molecules, and causes endothelial dysfunction in many pathophysiological conditions (Zhang et al., 2009). The p65 heterodimer, which is expressed in endothelial cells, is one of the most abundant NF-κB family members. A previous study investigated the association between enhanced nuclear transcription of the p65 subunit and thickening of the intima of the endothelium of human atherosclerotic plaques (Yerneni et al., 1999).

Accumulating evidence suggests that activated JNK and p38 play critical roles in cardiac injury and heart failure, as well as in many other cardiovascular diseases (Missiou et al., 2010; Ai et al., 2016; Singh et al., 2017). The JNK and p38 signaling pathway is a critical signal transduction pathway that participates in endothelial survival and injury (Kawano et al., 2014). A recent study demonstrated (Zhang et al., 2015) that the protective effects of Rb1 are attributable to suppression of the JNK and p38 MAPK signaling pathway in hydrogen Ang II-induced human aortic smooth muscle cell (HASMC) damage. Therefore, whether Rb1 attenuates JNK and p38 pathway activity to suppress TNF-α-induced human umbilical vein endothelial cell (HUVEC) injury must be verified.

**FIGURE 1:**
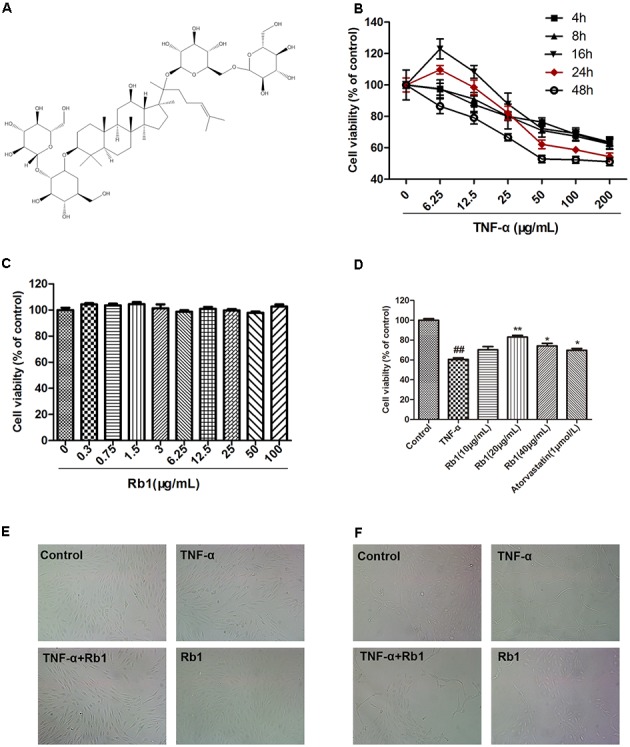
Ginsenoside Rb1 protected human umbilical vein endothelial cells (HUVECs) against tumor necrosis factor-α (TNF-α)-induced injury. **(A)** The chemical structure of ginsenoside Rb1. **(B)** HUVECs were treated with TNF-α (6.25, 12.5, 25, 50, 100, and 200 μg/mL) for different time intervals (4, 8, 16, 24, and 48 h). Cell viability was detected by MTT assay. **(C)** Ginsenoside Rb1 treatment alone has no toxic effect on the cell viability of HUVECs. **(D)** HUVECs were pretreated with Rb1 (10, 20, and 40 μg/mL) or atorvastatin (1 μmol/L) for 8 h and then incubated with TNF-α for 24 h. Cell viability was detected by MTT assay. **(E)** HUVECs were pretreated with Rb1 (20 μg/mL) for 8 h and then incubated with TNF-α for 24 h. Morphological images were obtained using an inverted microscope connected to a digital camera. **(F)** Matrigel was added to each well, and HUVECs were cultured and exposed to TNF-α for 24 h after incubation with Rb1 (20 μg/mL) for 8 h. Morphological images of tube formation in HUVECs were captured using the inverted microscope. Data were expressed as the mean ± SD of three independent experiments. ^##^*P* < 0.01 vs. control group; ^∗^*P* < 0.05, ^∗∗^*P* < 0.01 vs. TNF-α treatment group.

Ginseng is a prominent herbal drug that has been used in Asian countries for 1000s of years. It is also one of the most extensively used botanical products in Western society (Wu et al., 2011). Ginsenosides are the crucial active constituents of ginseng and are thus responsible for its pharmacological effects (Chen W. et al., 2016). The ginsenoside Rb1 (**Figure 1A**)—which is derived from the roots, stems, and leaves of Araliaceae ginseng; the roots and leaves of Panax ginseng; and the leaves and stems of Gynostemma from the Cucurbitaceae family—is a bioactive ingredient that exerts many beneficial effects (Guo et al., 2017). The most attractive characteristic of Rb1 is its ability to modulate the cardiovascular system, wherein it can regulate arrhythmias and promote the release of catecholamines to invigorate the heart (Wang et al., 2008). In addition, Rb1 can regulate the expression of actin cytoskeleton-related proteins to protect against AS and has been reported to inhibit inflammation and ameliorate insulin resistance under ER stress conditions (Chen W. et al., 2016; Hwang et al., 2016). However, no studies have highlighted the protective effects of Rb1 on TNF-α-induced endothelial cell injury, and the mechanisms underlying these effects have not yet been elucidated.

Thus, the present study aimed to investigate the protective effects of ginsenoside Rb1 and the molecular mechanisms underlying its effects on HUVECs subjected to TNF-α-induced injury. The focus was specifically on the relationship between Rb1 and the JNK and p38 pathway. Rb1 has strong anti-inflammatory effects and can directly inhibit NF-κB transcription, reduce inflammatory factor production and exert anti-apoptotic effects in endothelial cells upon TNF-α treatment. The results of the study indicate that ginsenoside Rb1 is a good drug candidate for developing agents that can attenuate the development of AS.

## Materials and Methods

### Reagents

Ginsenoside Rb1 (molecular weight = 1109, molecular structure shown in **Figure 1**) with a purity greater than 98% was obtained from Shanghai Winherb Medical S&T Development (Shanghai, China). Human recombinant TNF-α was purchased from Sigma–Aldrich (St. Louis, MO, United States), and VascuLife medium was obtained from LifeLine Cell Technology (Frederick, MD, United States). The cell culture materials were acquired from Gibco (Grand Island, NY, United States). The fluorescent dye (JC-1) kits and 3-(4,5-dimethylthiazol-2-yl)-2,5-diphenyltetrazolium bromide (MTT) assay kit used herein were acquired from Enzo Life Sciences (Farmingdale, NY, United States). The Annexin V-propidium iodide (PI) double-staining kit used for flow cytometry was acquired from Invitrogen (Carlsbad, CA, United States). The terminal deoxynucleotidyl transferase biotin-dUTP nick end labeling (TUNEL) assay kit and the kits used to measure the levels of intercellular cell adhesion molecule-1 (ICAM-1) and vascular cell adhesion molecule-1 (VCAM-1) release and IL-1β, interleukin-6 (IL-6), VEGF, MMP-2 and MMP-9 levels were obtained from Roche Diagnostics GmbH (Mannheim, Germany). Detection kits for ROS, MDA and the activities of SOD, CAT, and GSH-Px were obtained from Nanjing Jiancheng Bioengineering Institute (Nanjing, China). All antibodies and cell nuclear protein extraction kits were obtained from Santa Cruz Biotechnology (Santa Cruz, CA, United States). Western blot assay kits were acquired from Pierce Biotechnology (Rockford, IL, United States). The JNK and p38 activator anisomycin (AM) was purchased from Selleckchem (Houston, TX, United States). Atorvastatin calcium, dimethyl sulfoxide (DMSO), collagenase I and other chemicals were obtained from Sigma–Aldrich (St. Louis, MO, United States).

### Cell Culture and Treatment

In accordance with national ethics legislation, the umbilical cords used in the study were donated by the Maternal and Child Care Service Centre, Beijing, China. HUVECs were obtained from fresh human umbilical veins with 0.1% collagenase I, as previously described (Yang et al., 2017). Briefly, the cells were cultured in VascuLife medium containing streptomycin (100 μg/mL) and penicillin (100 U/mL). HUVECs from passages 2 to 4 were used in the experiments.

For all trials, the cells were seeded at a proper density, which was determined by our experimental protocol, and cultured for 36 h before treatment. The cells were assigned to one of the following four experimental groups: (1) the control group, (2) the TNF-α pretreatment group, (3) the Rb1 and TNF-α treatment group, or (4) the Rb1 pretreatment group.

### Analysis of Cell Viability

Cell viability was assessed by MTT assay. Briefly, HUVECs were seeded in 96-well gelatin-coated plates. The cells were first preconditioned with different concentrations of TNF-α, and cell viability was determined at 2, 4, 8, 16, 24, and 48 h after treatment initiation to determine the optimal molding condition. Then, 1 mg/mL MTT solution was added to each well, and the cells were incubated for 4 h at 37°C. The supernatant was subsequently removed and replaced with 100 μL of DMSO, after which the cells were shaken for 2 min. The absorbance was measured at 570 nm on a microplate reader (SpectraFluor, Tecan, Austria). Cell viability was expressed as a percentage of the control. After the optimal molding condition was determined, the cells were pretreated with different doses of Rb1 (10, 20, and 40 μg/mL) for 8 h.

### Flow Cytometry Detection of Apoptosis

The proportions of viable and apoptotic cells in each treatment group were measured with Annexin V-PI double-staining kits (Invitrogen, Carlsbad, CA, United States) by flow cytometry, as previously described (Ai et al., 2015). Briefly, HUVECs were cultured on 6-well collagen-coated plates. After being preincubated with 20 μg/mL Rb1 for 8 h, the HUVECs were washed with PBS buffer and cultured with 50 ng/mL TNF-α for 24 h. HUVECs from each group were subsequently harvested before being washed twice with ice-cold PBS and then incubated with 100 μL of 1× Annexin V work solution supplemented with PI (1 μg/mL final concentration) in the dark for 15 min at room temperature. The cells were then treated with 400 μL of 1× binding buffer before being vortexed briefly. The cells were subsequently analyzed by a FACSCalibur flow cytometer (Becton, Dickinson and Company, San Diego, CA, United States).

### Measurement of Mitochondrial Transmembrane Potential ΔΨm (MMP)

Changes in mitochondrial membrane potential were investigated by JC-1 (Enzo Life Sciences International, United States) staining. Different groups of HUVECs were seeded in 6-well collagen-coated plates. After being pretreated with 20 μg/mL Rb1 for 8 h and then incubated with 50 ng/mL TNF-α for 24 h, the HUVECs were washed with phosphate buffered saline (PBS) and then incubated with 2 μM JC-1 in the dark for 30 min at 37°C. The cells were then washed twice with PBS before being imaged using a fluorescence microscope (Molecular Devices, United States).

### TUNEL Staining in HUVECs

A TUNEL kit was used to measure DNA fragmentation in the apoptotic cell population according to the manufacturer’s instructions. Briefly, HUVECs were cultured on 24-well collagen-coated plates for 36 h. After routine pretreatment with 20 μg/mL Rb1 for 8 h, followed by treatment with 50 ng/mL TNF-α for 24 h, the cells were fixed with 4% paraformaldehyde solution in PBS, as recommended by the manufacturer. A methanol solution containing 0.3% H_2_O_2_ was used to block cellular endogenous peroxidase activity for 30 min at room temperature, after which the cells were treated with a solution comprising 0.1% Triton X-100 and 0.1% sodium citrate for permeabilization. Each sample was subsequently incubated with TUNEL reaction mixture for 60 min at 37°C. After being rinsed with PBS, the cells were imaged using a fluorescence microscope (Leica DM4000, Germany), and the percentage of TUNEL-positive cells was expressed in accordance with previously described methods (Wang et al., 2013).

### Measurement of Oxidative Stress and Inflammation Markers by ELISA

After the cells were treated with the appropriate drugs and vehicles, the levels of ROS, MDA, IL-1β, IL-6, VEGF, MMP-2 and MMP-9 and the activities of SOD, CAT, and GSH-Px were detected using the appropriate ELISA kits as recommended by the manufacturer.

### Detection of NF-κB Transcription by Immunofluorescence

For intracellular NF-κB staining, HUVECs were seeded in a 24-well plate. Different groups of HUVECs were pretreated with Rb1 or TNF-α before being harvested and then fixed with 4% paraformaldehyde in PBS for 10 min at room temperature. After incubating with 100% methanol for 10 min at -20°C, the cells were treated with 0.1% sodium citrate and 0.1% Triton X-100 for permeabilization. Then, 1% BSA was added to the wells to block the previous reaction for 1 h, after which the cells were incubated with primary antibodies against NF-κB overnight at 4°C. After being washed with PBS, the cells were incubated with specific secondary antibodies conjugated with fluorescein isothiocyanate (FITC) fluorochromes (Alexa Fluor, Molecular Probes, Life Technologies) for 30 min at room temperature. Images of the stained cells were obtained under a fluorescence microscope.

### Western Blot Analysis

After the cells were treated with the appropriate drugs and vehicles, the nuclear proteins and whole-cell lysates were obtained using cell nuclear and cytoplasmic protein extraction kits. The cells were treated with phosphatase inhibitor cocktail and protease inhibitor cocktail (CoWin Bioscience Co., Ltd., Beijing, China), and a BCA Protein Assay Kit was used to detect the protein concentrations (Pierce Corporation, Rockford, IL, United States) according to the manufacturer’s instructions. Western blot analysis was performed as previously described (Meng et al., 2014). Briefly, equal amounts of protein from different samples were resolved by electrophoresis on 8–12% sodium dodecyl sulfate polyacrylamide gels (SDS–PAGE) before being transferred onto nitrocellulose membranes in Tris-glycine buffer at 100 V for 50 min in an ice box. The membranes were subsequently blocked with TBST containing 5% (w/v) non-fat milk powder and then incubated with the appropriate primary antibodies (1:200) overnight at 4°C. The membranes were then incubated with the appropriate secondary HRP-conjugated antibodies (1:1000) for 2 h on a shaking table. After being rinsed with TBST three times for 45 min each, the blots were developed using enhanced chemiluminescence solution for 5 min, after which they were scanned with Image Lab software (Bio-Rad, United States). β-actin or Lamin B expression levels were also measured to certify that the proteins were loaded equally, and Gel-Pro Analyzer software was used to calculate protein expression levels, which were expressed as percentages of the control.

### Statistical Analysis

Data are expressed as the means ± standard deviation (SD) of at least three independent experiments. Differences between groups were assessed by one-way ANOVA followed by the Student-Newman-Keuls test using GraphPad Prism 5.0 software. Statistical significance was defined as *P* < 0.05.

## Results

### Ginsenoside Rb1 Protects HUVECs against TNF-α-Induced Cell Death

We established the TNF-α-stimulated HUVEC damage model by incubating HUVECs with different concentrations of TNF-α (0, 6.25, 12.5, 25, 50, 100, and 200 ng/mL). Cell viability was detected at 2, 4, 8, 12, 24, and 48 h post-treatment initiation using MTT assay and was expressed as a percentage of the control. As shown in **Figure 1B**, TNF-α treatment resulted in additional time-and dose-dependent decreases in cell viability in treated cells compared with that of control cells. Specifically, treatment with 50 ng/mL TNF-α for 24 h reduced cell viability by approximately 40% in the former group compared with the latter group.

To determine the best condition under which Rb1 can protect against TNF-α-induced HUVEC injury, we first determined whether Rb1 has cytotoxic or proliferative effects on HUVECs. We noted no changes in cell viability after HUVECs were treated with the indicated Rb1 concentrations (0.3, 0.75, 1.5, 3.125, 6.25, 12.5, 25, 50, and 100 μg/mL) for 8 h. We therefore concluded that Rb1 administration does not change cellular viability (**Figure 1C**). We then pretreated HUVECs with Rb1 at the indicated concentrations (10, 20, and 40 μg/ml) for 8 h, after which we incubated the cells with TNF-α for 24 h, using atorvastatin calcium as a positive control. The results indicated that 20 and 40 μg/mL Rb1 had significant cytoprotective effects and that 20 μg/mL was the most effective Rb1 concentration (**Figures 1D,E**). In addition, TNF-α clearly induced the formation of tubes, whereas Rb1 pretreatment significantly inhibited tube formation (**Figure 1F**).

### Ginsenoside Rb1 Attenuates TNF-α-Induced Inflammation in HUVECs

**FIGURE 2:**
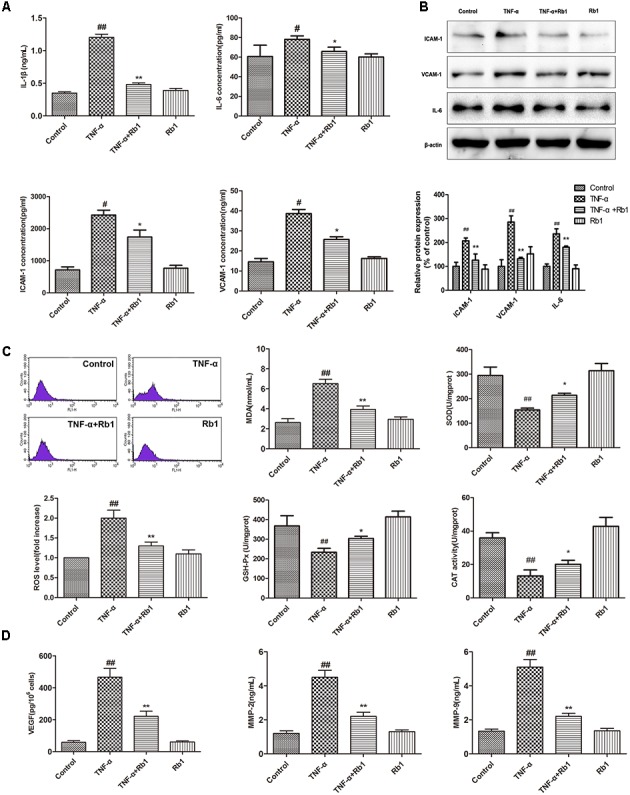
Ginsenoside Rb1 could suppress TNF-α-induced inflammation in HUVECs. HUVECs were treated with TNF-α for 24 h after incubation with Rb1 (20 μg/mL) for 8 h. **(A)** The levels of IL-1β, ICAM-1, VCAM-1, and IL-6 were measured by ELISA. **(B)** The protein expression levels of ICAM-1, VCAM-1, and IL-6 were determined by western blot and quantified using densitometric analysis. **(C)** ROS levels were detected by flow cytometry, and the levels of MDA and the activities of SOD, CAT, and GSH-Px were detected by respective assay kits. **(D)** The levels of VEGF, MMP-2, and MMP-9 in HUVECs were detected by ELISA. Data were expressed as the mean ± SD of three independent experiments. ^##^*P* < 0.01 vs. control group; ^∗^*P* < 0.05, ^∗∗^*P* < 0.01 vs. TNF-α treatment group.

To confirm the hypothesis that Rb1 protects HUVECs by exerting anti-oxidative and inflammatory effects, we directly measured the levels of ROS, MDA, IL-1β, IL-6, VEGF, MMP-2 and MMP-9 and the activities of SOD, CAT, and GSH-Px. As shown in **Figures 2A–D**, the levels of ROS, MDA, IL-1β, IL-6, VEGF, MMP-2 and MMP-9 were significantly increased and the activities of SOD, CAT, and GSH-Px were significantly decreased in the TNF-α-treated group compared with the control group; these results indicate that TNF-α exerts its cytotoxic effects by inducing oxidative stress and inflammation. Pretreatment with Rb1 effectively attenuated the abovementioned factors in the TNF-α and Rb1 treatment group. However, treatment with Rb1 alone had no effect on the levels of the indicated oxidative stress and inflammatory markers.

Furthermore, western blot analysis also showed that ICAM-1, VCAM-1, and IL-6 expression was induced by TNF-α treatment, effects that were inhibited by Rb1 administration (**Figure 2B**). Taken together, the aforementioned results indicate that Rb1 protects against TNF-α-induced HUVEC injury through its anti-oxidative and inflammatory effects.

### Ginsenoside Rb1 Ameliorates TNF-α-Induced Apoptosis in HUVECs

We assessed apoptotic cell death to determine the type of cell death that is induced by TNF-α and to elucidate the mechanism underlying the protective effects of ginsenoside Rb1 on HUVECs.

**FIGURE 3:**
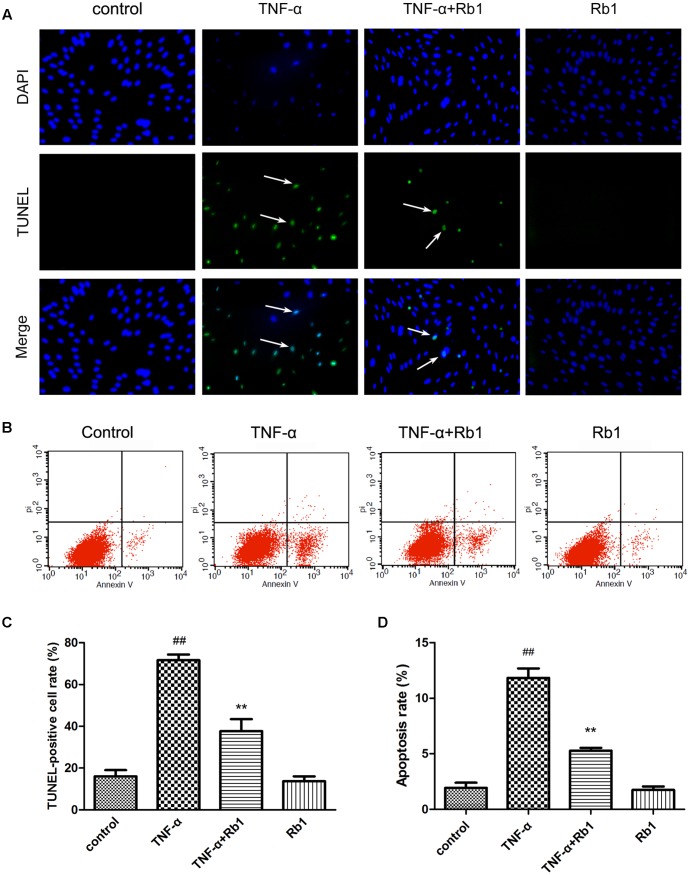
Ginsenoside Rb1 could inhibit TNF-α-induced apoptosis. HUVECs were subjected to TNF-α for 24 h after being pretreated with Rb1 (20 μg/mL) for 8 h. **(A,B)** Apoptosis of HUVECs was assessed by TUNEL staining and Annexin V/PI staining. **(C)** Quantitative analysis of TUNEL-positive cell rate. **(D)** Quantitative analysis of apoptosis rate. Data were expressed as the mean ± SD of three independent experiments. ^##^*P* < 0.01 vs. control group; ^∗^*P* < 0.05, ^∗∗^*P* < 0.01 vs. TNF-α treatment group.

TUNEL staining was used in the current study (**Figure 3A**). The rate of TUNEL positivity was significantly higher in the TNF-α-treated group than in the control group (**Figure 3C**), indicating that treatment with TNF-α caused DNA fragmentation in HUVECs. This phenomenon was significantly ameliorated by pretreatment with Rb1 (**Figure 3C**). These results suggest that Rb1 can protect HUVECs from TNF-α-induced apoptosis.

Annexin V-PI double-staining is a well-accepted method for analyzing early-stage apoptosis (Sun et al., 2013). Thus, to assess the protective effects of Rb1 against TNF-α-induced cell death, we performed Annexin V/PI double staining to quantify apoptosis in cells treated with the abovementioned agents. The percentage of apoptotic cells was significantly increased in the TNF-α-treated group compared with that in the control group (**Figures 3B,D**). However, Rb1 pretreatment reversed the abovementioned increases in apoptotic cell numbers. Rb1 treatment alone had no effect on HUVEC apoptosis rates.

### Ginsenoside Rb1 Alleviates Mitochondrial Membrane Depolarization in HUVECs

**FIGURE 4:**
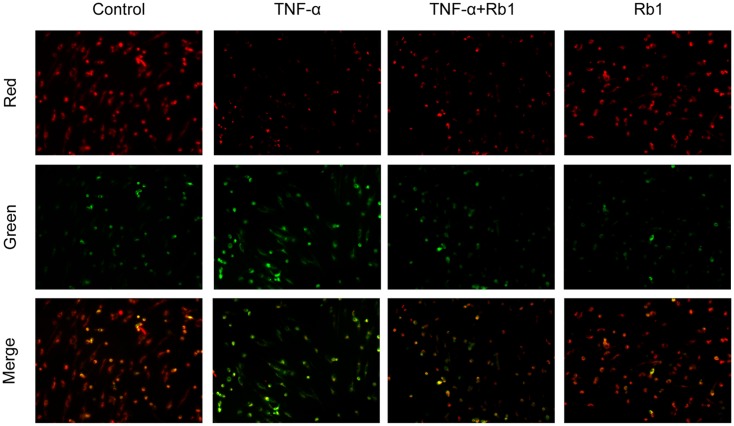
Effects of ginsenoside Rb1 on mitochondrial transmembrane potential. HUVECs were pretreated with Rb1 (20 μg/mL) for 8 h and then incubated with TNF-α for 24 h. Mitochondrial membrane potential was detected by JC-1 staining, which was visualized by fluorescence microscopy. The images are representative of three experiments.

Tumor necrosis factor-α-induced endothelial cell injury may lead to opening of the mitochondrial permeability transition pore (mPTP) in the inner mitochondrial membrane, resulting in mitochondrial membrane depolarization and pro-apoptotic substance release (Takabe et al., 2010). To determine whether Rb1 mitigates TNF-α-induced mitochondrial injury, we used JC-1 to determine the mitochondrial membrane potential. The results of the experiment suggested that TNF-α caused Dym depolarization, an effect that was significantly attenuated by pretreatment with Rb1 (**Figure 4**). Ginsenoside Rb1 treatment alone had no effect on mitochondrial membrane potential in HUVECs. These results show that Rb1 can inhibit mitochondrial damage in HUVECs.

### Ginsenoside Rb1 Modulated Apoptosis-Related Protein Expression in TNF-α-Induced HUVECs

**FIGURE 5:**
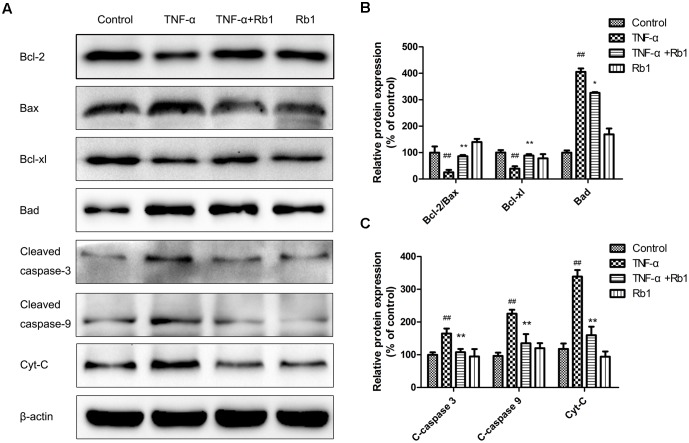
Effects of ginsenoside Rb1 on apoptosis-related protein expression in TNF-α-treated HUVECs. HUVECs were exposed to TNF-α for 24 h after incubation with ginsenoside Rb1 (20 μg/mL) for 8 h. **(A)** The levels of Bcl-2/Bax, Bcl-xl/Bad, cleaved caspase-9 and cleaved caspase-3 were determined by western blot. **(B)** Quantitative analysis of Bcl-2/Bax expression ratio and Bcl-xl and Bad expression levels. **(C)** Quantitative analysis of cleaved caspase-3, cleaved caspase-9, and Cyt-c expression. Data were expressed as the mean ± SD of three independent experiments. ^##^*P* < 0.01 vs. control group; ^∗^*P* < 0.05, ^∗∗^*P* < 0.01 vs. TNF-α treatment group.

To illuminate the mechanism underlying the effects of ginsenoside Rb1 on TNF-α-induced apoptosis, we investigated apoptotic protein expression in HUVECs. Bax, Bcl-2, Bad, and Bcl-xl, which are Bcl-2 protein family members, play a key role in regulating endotheliocyte apoptosis (Yang et al., 2017). As shown in **Figure 5**, treatment with TNF-α down-regulated Bcl-2 protein expression and up-regulated Bax protein expression. These effects were reversed by pretreatment with Rb1, which increased the Bcl-2/Bax ratio and Bcl-xl expression and significantly decreased Bad expression. Moreover, TNF-α treatment enhanced cleaved caspase-3, 9 expression; however, Rb1 pretreatment inhibited these increases. Cytochrome-c (Cyt-c), a key mitochondrial protein that serves as an index of apoptosis (Meng et al., 2013), was released into the cytoplasm when the cells were exposed to TNF-α, an effect that was inhibited by treatment with Rb1. Taken together, these results suggest that Rb1 can increase anti-apoptotic protein expression and reduce pro-apoptotic protein expression to protect against endotheliocyte apoptosis.

### Ginsenoside Rb1 Down-Regulates NF-κB Nuclear Transcription

**FIGURE 6:**
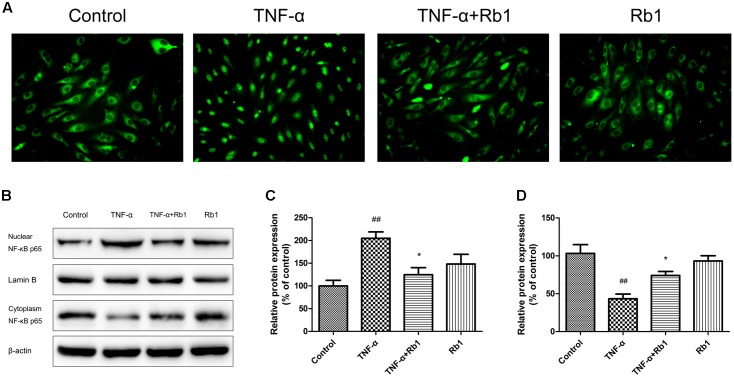
The protective effects of ginsenoside Rb1 on TNF-α-induced injury in HUVECs are NF-κB-dependent. HUVECs were incubated with ginsenoside Rb1 (20 μg/mL) for 8 h prior to treatment with TNF-α for 24 h. **(A)** The localization of p65 in HUVECs was detected by immunofluorescence and visualized by fluorescence microscopy. **(B)** Western blot analysis was performed to examine nuclear and cytoplasmic NF-κB p65 expression. **(C)** Densitometric analysis was performed to quantify nuclear NF-κB p65 protein levels. **(D)** Densitometric analysis was performed to quantify cytoplasmic NF-κB p65 protein levels. Data were expressed as the mean ± SD of three independent experiments. ^##^*P* < 0.01 vs. control group; ^∗^*P* < 0.05, ^∗∗^*P* < 0.01 vs. TNF-α treatment group.

NF-κB plays a key role in the inflammatory cascade when exposed to different stimuli, and its transcription may ultimately leads to apoptotic cell death. To identify the signaling pathways that facilitate the anti-apoptotic effects of Rb1, we examined the kinetics of NF-κB p65, which is regulated by Rb1, via microscopy and western blotting. As shown in **Figure 6A**, TNF-α treatment resulted in significant NF-κB transcription in TNF-α-treated cells compared with control cells; however, Rb1 administration significantly inhibited TNF-α-induced NF-κB transcription in the corresponding group of cells compared with the abovementioned groups of cells. The abovementioned TNF-α-induced increases in NF-κB expression in the cell nucleus and decreases in NF-κB expression in the cytoplasm were verified by western blotting (**Figures 6B–D**). These results indicate that the anti-inflammatory effects of Rb1 are associated with its ability to regulate NF-κB.

### Ginsenoside Rb1 Inhibited NF-κB Signaling by Suppressing the p-38 and JNK Pathway

**FIGURE 7:**
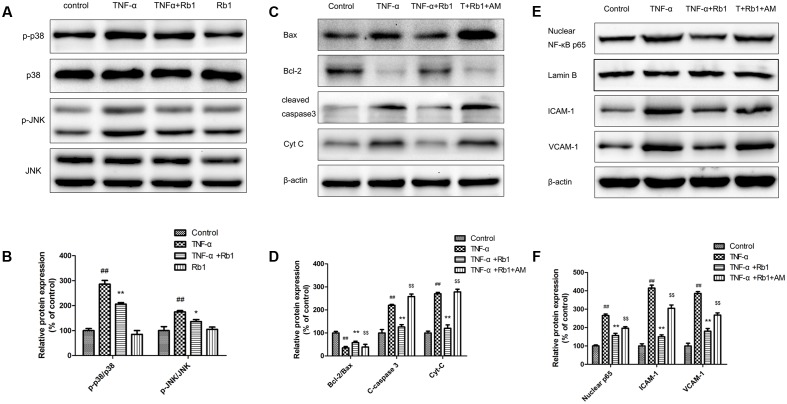
The protective effects of ginsenoside Rb1 on TNF-α-induced injury in HUVECs are p38 and JNK-pathway dependent. HUVECs were incubated with ginsenoside Rb1 (20 μg/mL) for 8 h before treatment with TNF-α for 24 h. **(A)** The expression levels of phosphorylated and total JNK and p38 were detected by western blot. **(C,E)** Ginsenoside Rb1 suppressed NF-κB nuclear translocation and regulated apoptosis-related protein expression by inhibiting the JNK and p38 pathway. **(B,D,F)** Densitometric analysis was performed to quantify protein expression levels. Data were expressed as the mean ± SD of three independent experiments. ^##^*P* < 0.01, vs. control group; ^∗^*P* < 0.05, ^∗∗^*P* < 0.01 vs. TNF-α treatment group. ^$^*P* < 0.05, ^$$^*P* < 0.01, vs. TNF-α and ginsenoside Rb1 groups.

An important signal transduction pathway, the p-38 and JNK pathway plays a pivotal role in regulating gene expression. To identify the signaling pathways that facilitate the anti-inflammatory and anti-apoptotic effects of Rb1, we examined the relationship among Rb1, p-38 and JNK activity and NF-κB activation. The results of the examination (**Figures 7A,B**) showed that Rb1 decreased TNF-α-induced JNK and p38 phosphorylation, suggesting that the JNK and p38 pathway plays a pivotal role in facilitating the effects of Rb1.

To confirm the effects of the JNK and p38 pathway, we pretreated HUVECs with AM, a widely known phosphorylated-JNK and p38 activator, for 1 h before the cells were treated with Rb1 and incubated with TNF-α. As shown in **Figures 7C,D**, the p-JNK/JNK and p-p38/p38 ratios were increased in TNF-α-treated cells compared with control cells. Pretreatment with Rb1 reversed the effects of TNF-α, while treatment with the JNK and p38 pathway activator abolished the protective effects of Rb1. Nuclear NF-κB, inflammatory factor and pro-apoptotic protein expression levels were significantly enhanced in TNF-α-treated cells compared with control cells. Pretreatment with Rb1 inhibited the expression of the abovementioned proteins, an effect that was reversed by AM. However, the expression levels of the anti-apoptotic proteins Bcl-2/Bax displayed the opposite trend, decreasing in response to TNF-α treatment and increasing in response to Rb1 treatment in the corresponding groups of treated cells compared with control cells (**Figures 7E,F**). Taken together, these results indicated that ginsenoside Rb1 inhibited TNF-α-induced apoptosis by down-regulating NF-κB signaling via JNK and p38 pathway suppression.

## Discussion

Endothelial injury is reported to be the initial step in the pathogenesis of most cardiovascular diseases, as well as the development of AS (Libby, 2012). It is now believed that AS, which was previously attributed to enhanced lipid deposition, results mainly from a series of inflammatory processes and that endothelial injury represents the initial stage of chronic vascular lesion formation (Stoll and Bendszus, 2006; Libby, 2012; Hamlat-Khennaf et al., 2017). Ginsenoside Rb1, a main constituent of the root of Panax ginseng, has various pharmacological effects, including anti-inflammatory and anti-apoptotic effects (Park et al., 2005). In the present study, we obtained the first evidence that ginsenoside Rb1 protects HUVECs against TNF-α-induced inflammatory injury and apoptosis and further explored the signaling pathway that facilitates this effect.

Considerable evidence indicates that TNF-α plays a major role in the initiation of an innate immune response involved in triggering and/or amplifying local inflammatory responses (Kleinbongard et al., 2011; Wang et al., 2014), and the model of TNF-α-induced endothelial cell inflammation-related damage is widely used to mimic the effects of inflammation-induced stress and injury (Wang et al., 2014). We found that TNF-α could reduce the cell viability of HUVECs in dose- and time-dependent manners. The cell viability of HUVECs decreased by approximately 40% when treated with TNF-α (50 ng/mL) for 24 h (**Figure 1**). This condition was considered the best for apoptosis induction; thus, we elected to evaluate the protective effects of ginsenoside Rb1 on endothelial cells. Our results showed that pre-incubation with ginsenoside Rb1 could significantly improve cell viability. We subsequently evaluated the protective and cytotoxic effects of Rb1 (**Figure 1**).

Endothelial cells are extremely susceptible to the effects of oxidative stress, pro-inflammatory cytokines and adhesion molecules (Kleinbongard et al., 2010). Our data demonstrated that Rb1 suppressed the levels of ROS, MDA, IL-1β, IL-6, VEGF, MMP-2 and MMP-9 and increased the activities of SOD, CAT, and GSH-Px. Additionally, we found that ginsenoside Rb1 inhibited TNF-α-induced VEGF production, subsequently inhibiting tube formation. Moreover, inflammation is considered to be a key factor in AS development because of its principle role in triggering endothelial cell apoptosis (Wu et al., 2011). Cleaved caspase-3, the activated form of caspase-3, a pro-apoptotic marker, initiates apoptosis. The findings of the present study indicated that TNF-α enhanced the level of cleaved caspase-3 in HUVECs; however, Rb1 pretreatment significantly inhibited this increase. Increases in inflammatory factor may disrupt mitochondrial membrane potential and initiate opening of the MPTP under pathological conditions (Ghosh et al., 2016). Our results indicated that Rb1 strongly attenuated TNF-α-induced mitochondrial damage. Sustained MPTP opening and membrane potential depolarization may lead to the release of Cyt-c, a critical mitochondrial death factor. These changes induce activation of caspase-9 and caspase-3 and ultimately apoptosis. However, Bcl-2, which resides in the mitochondrial membrane and forms heterodimers with Bax, may block the mitochondrial apoptosis pathway (Solesio et al., 2013). Our results demonstrated that TNF-α triggers the mitochondrial apoptosis pathway by up-regulating the levels of cytoplasmic Cyt-c, cleaved caspase-3 and cleaved caspase-9 and disrupting the balance between Bcl-2 and Bax. These data indicated that Rb1 pretreatment enhanced anti-apoptotic protein expression but reduced pro-apoptotic protein expression. Taken together, the results of our study clearly indicate that ginsenoside Rb1 protects HUVECs from TNF-α-induced apoptosis by suppressing the mitochondrial apoptotic pathway.

NF-κB activation is indispensable for the regulation of critical factors responsible for the inflammatory reactions that play crucial roles in AS (Tak and Firestein, 2001). The present study was designed to elucidate the effects of Rb1 on NF-κB transcription. The results demonstrated that NF-κB/p65 was activated and translocated into the nucleus after TNF-α stimulation; however, Rb1 pretreatment could reverse this effect, results consistent with those of the western blot analysis. These observations clearly indicate that Rb1 specifically attenuated TNF-α-induced NF-κB translocation, which is reported to be the principle event responsible for the inflammatory response.

Considerable evidence indicates that MAPK participates in AS (Muslin, 2008; Wagner and Nebreda, 2009). The three subunits of the MAPK family, namely, ERK, JNK, and p38, are frequently associated with AS processes in both humans and mice, and their signaling pathways are extensively involved in apoptosis and inflammation initiation in different cell types (Zhan et al., 2003; Han et al., 2010). ERK, JNK, and p38 share 60–70% structural identity with one another but exert a variety of cellular effects because of differences in their sizes and their activation loop sequences (King et al., 2009). Consistent with the results of previous studies (Cheng et al., 2013; Kanaji et al., 2013), our results showed that the phosphorylation levels of all the MAPK family subunits were increased in TNF-α-induced endothelial cell injury. However, only JNK and p38 activities were significantly inhibited by Rb1 pre-incubation. Importantly, AM, a specific JNK and p38 activator, then largely abolished the protective effects of Rb1 treatment. Collectively, these results clearly indicate that the protective effects of ginsenoside Rb1 are mediated by its suppression of the p38 MAPK and JNK signaling pathway.

## Conclusion

**FIGURE 8:**
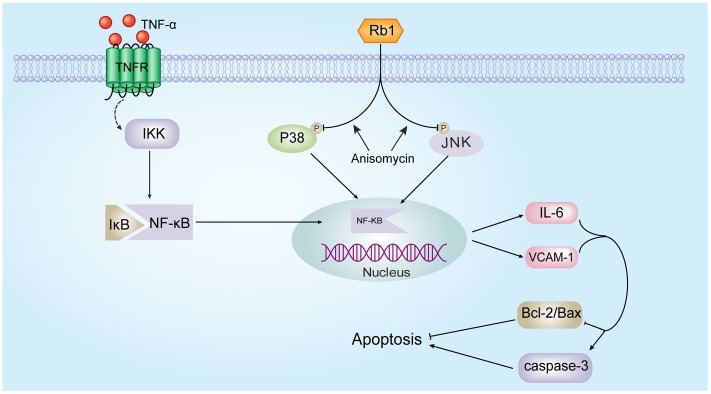
Schematic of the mechanism by which ginsenoside Rb1 prevents TNF-α-induced inflammation in HUVECs.

Our results represent the first evidence that ginsenoside Rb1 significantly ameliorates TNF-α-induced HUVEC injury. This protective effect was associated with the inactivation of a series of inflammatory reactions and the inhibition of NF-κB translocation, a phenomenon that was JNK- and p38 MAPK-dependent (**Figure 8**). If these effects of ginsenoside Rb1 are validated in animal studies and clinical trials, it might be a promising agent for the prevention and treatment of AS.

## Author Contributions

PZ, GS, and XS contributed to the conception of the study. PZ, SL, and YL contributed significantly to analysis and manuscript preparation. PZ, SW, and KY performed the data analyses and wrote the manuscript. KY and YZ helped perform the analysis with constructive discussions.

## Conflict of Interest Statement

The authors declare that the research was conducted in the absence of any commercial or financial relationships that could be construed as a potential conflict of interest.
